# Seawater-Cultured *Botryococcus braunii* for Efficient Hydrocarbon Extraction

**DOI:** 10.1371/journal.pone.0066483

**Published:** 2013-06-14

**Authors:** Kenichi Furuhashi, Kiyotaka Saga, Shigeru Okada, Kenji Imou

**Affiliations:** 1 Laboratory of Biological and Mechanical Engineering, Graduate School of Agricultural and Life Sciences, the University of Tokyo, Yayoi, Bunkyo-ku, Japan; 2 Laboratory of Aquatic and Natural Products Chemistry, Graduate School of Agricultural and Life Sciences, the University of Tokyo, Yayoi, Bunkyo-ku, Japan; National University of Singapore, Singapore

## Abstract

As a potential source of biofuel, the green colonial microalga *Botryococcus braunii* produces large amounts of hydrocarbons that are accumulated in the extracellular matrix. Generally, pretreatment such as drying or heating of wet algae is needed for sufficient recoveries of hydrocarbons from *B. braunii* using organic solvents. In this study, the Showa strain of *B. braunii* was cultured in media derived from the modified Chu13 medium by supplying artificial seawater, natural seawater, or NaCl. After a certain period of culture in the media with an osmotic pressure corresponding to 1/4-seawater, hydrocarbon recovery rates exceeding 90% were obtained by simply mixing intact wet algae with *n*-hexane without any pretreatments and the results using the present culture conditions indicate the potential for hydrocarbon milking.

**Highlights:**

Seawater was used for efficient hydrocarbon extraction from *Botryococcus braunii*. The alga was cultured in media prepared with seawater or NaCl. Hydrocarbon recovery rate exceeding 90% was obtained without any pretreatment.

## Introduction

Biomass energy has recently attracted considerable attention as a renewable energy source to address global warming, air pollution and depletion of fossil fuels. Biofuel production from microalgae is an example of biomass utilization that has a high rate of productivity per unit area [1]. *Botryococcus braunii*, a colony-forming green microalga, produces hydrocarbons at a high rate per unit of dry cell weight [2], [3]. There are three races of *B. braunii*, which are classified into A, B, L race according to the type of hydrocarbons produced [4]. The B race of *B. braunii* produces C_30_ to C_37_ isoprenoid hydrocarbons (botryococcenes) and small amounts of methyl squalenes [5]. These hydrocarbons generally vary between 10 to 40% of the dry cell weight in different strains and under variable culture conditions [6], [7]. In terms of thermal values, hydrocarbons are more attractive as a biofuel for jet engines than lipids accumulated by other microalgae that are usually derivatives of fatty acids. Therefore *B. braunii* is one of the most promising microalgae as a renewable energy source. Moreover, most of the hydrocarbons (90–95%) of this alga are stored in an extracellular matrix composed of a polymer core of aldehydes [8]. This also is advantageous for biofuel production because cell breaking is not necessary to recover the hydrocarbons. Actually droplets of hydrocarbons extruded from the extracellular matrix can be seen under a microscope as far as algal colonies are placed on a slide glass in a single layer and are mechanically compressed with a cover glass. It is, however, difficult to recover hydrocarbons from wet *B. braunii* cells by simple compression or solvent extraction. In a study by Frenz et al., a hydrocarbon recovery rate of less than 1% was observed when *n*-hexane was mixed with intact wet algae [9]. Generally, pretreatment such as drying or heating of wet algae is needed for *B. braunii* to achieve a hydrocarbon recovery rate of over 90% using organic solvents [10], [11]. This discrepancy between the location of hydrocarbons at outside of cells and the difficulty in hydrocarbon recovery from wet algal colonies might be caused by the unique structure of colonies of *B. braunii*. In this context, Weiss et al. reported the colony organization in *B. braunii* using the quick freeze deep-etch electron microscopy technique [12]. They suggested the possibility that the outer structure called “retaining wall” composed of mainly carbohydrate serves to sequester the liquid hydrocarbons within the colony. Therefore the pretreatment such as drying or heating may change structure or property of the extracellular matrix in the algal colonies, and let extrusion of hydrocarbons from the colonies easier as the result.

*B. braunii* is originally a freshwater alga [13]. Its tolerance to salinity varies among the strains [14], [15]. When industrial scale culture is considered of the alga for hydrocarbon production, the use of seawater for culture medium would be more desirable compared to freshwater because the global abundance of seawater. The use of seawater may also reduce the risk of contamination by other freshwater-living organisms in the culture pond. In this context, a preliminary experiment was carried out using the Showa strain belonging to the B race of *B. braunii* to see if seawater can be used for its culture. It was cultured in each medium prepared with artificial seawater, in which salinity was adjusted to 1/1, 1/2, and 1/4 of seawater, respectively. Algal death was observed in the 1/1 and 1/2 media, but an increase in algal biomass was observed in the 1/4 medium. In addition to the survival of alga, it was found that hydrocarbon was extracted more readily from *B. braunii* cultured in the 1/4 seawater medium compared with those cultured in a freshwater medium.

High recovery of lipids from wet sample of *Chlamydomonas reinhardtii* by osmotic shock was reported [16]. In that study, wet algal cells were treated with relatively high concentrations of NaCl or sorbitol to give sufficient osmotic shocks. In contrast, our culture in 1/4 seawater medium may not give the alga such a severe osmotic shock because the algal biomass increased. Therefore this new finding may have a potentiality to do “milking” of hydrocarbons from living *B. braunii*.

There are two objectives in this work. One is to clarify how the length of the culture period in a medium of 1/4 concentration of artificial seawater impacts the hydrocarbon production and recovery rate. The other is to identify the effect of salinity on high hydrocarbon recovery through comparison between the 1/4 artificial seawater medium and culture media using natural seawater or NaCl.

## Materials and Methods

### 1. Culture of *B. braunii*

In this study, the Showa strain, which is classified in the B race was used. The Showa strain was cultured in four types of media. The first type was the modified Chu13 medium often used for *B. braunii* [17], [18]. The second type was the 1/4 artificial seawater medium (1/4 ASWM) prepared by diluting a commercial artificial seawater (Daigo’s Artificial Seawater SP for Marine Microalgae Medium, Wako Pure Chemical Industries) to 1/4 concentration of seawater with deionized water and adding salts such as KNO_3_, K_2_HPO_4_⋅3H_2_O and FeNaEDTA that are included in the modified Chu13 medium but not included in the artificial seawater (Table 1). The third type was natural seawater medium (1/4 NSWM). 1/4 NSWM was prepared in the same way as 1/4 ASWM. Instead of artificial seawater, natural seawater from the open ocean surrounding the Izu Islands in the Pacific was used after dilution to 1/4 concentration with deionized water. The components included in the modified Chu13 medium were similarly supplemented to 1/4 NSWM (Table 1). The fourth type was sodium chloride medium (NaCl medium). It was also basically the same as the modified Chu13 medium, but its osmotic pressure was adjusted to the same as that for the 1/4 artificial seawater medium by adding NaCl at a concentration of 8.80 g/L. The composition of each culture medium is shown in Table 1.

**Table 1:** Composition of each type of medium applied in this study.

Component (mg/L)	Modified Chu13	1/4 ASWM	1/4 NSWM	NaCl medium
KNO_3_	600	600^a^	600	600
MgSO_4_⋅7H_2_O	100			100
CaCl_2_⋅2H_2_O	54	331.5		54
K_2_HPO_4_⋅3H_2_O	52	52^a^	52	52
FeNaEDTA	10	10^a^	10	10
H_3_BO_3_	2.36	2.36^a^	2.36	2.36
MnSO_4_⋅H_2_O	1.54	1.54^a^	1.54	1.54
ZnSO_4_⋅7H_2_O	0.22	0.22^a^	0.22	0.22
CoSO_4_⋅7H_2_O	0.09	0.09^a^	0.09	0.09
CuSO_4_⋅7H_2_O	0.08	0.08^a^	0.08	0.08
Na_2_MoO_4_⋅2H_2_O	0.06	0.06^a^	0.06	0.06
NaCl		5186.75		8800
MgCl_2_⋅6H_2_O		2368.5		
Na_2_SO4		876.25		
KCl		149.25		
NaHCO_3_		42.75		
KBr		21.25		
Na_2_B_4_O_7_⋅10H_2_O		8.5		
SrCl_2_		3		
NaF		0.75		
LiCl		0.25		
KI		0.0175		
(NH_4_)_6_Mo_7_O_24_⋅3H_2_O		0.005		
AlCl_3_⋅6H_2_O		0.002		
FeCl_3_⋅6H_2_O		0.00125		
MnCl_2_⋅4H_2_O		0.0002		
Na_2_WO_4_⋅2H_2_O		0.00005		
Natural seawater			250 ml^b^	
Deionized water	1000 ml	1000 ml	750 ml	1000 ml

a Added to offset differences compared with the modified Chu13 medium.

b Two hundred and fifty milliliters of natural seawater taken directly from the open sea.

Algal cells were cultured in 1.5 L glass flasks containing each type of medium under the following conditions. Sterile air with 1.3% CO_2_ passing through a vent filter was continuously aerated into the flask. Illumination intensity was set to around 50 µmol/m^2^/s with a light-dark cycle of 12∶12 h and culture temperature was set at 25°C. The alga was repeatedly subcultured every ca. 35 days and these periods between subcultures were each treated as one culture period. The reason why we defined a cycle of 35 days as one culture period was because the alga reached the stationary phase at 35 days under the culture conditions and the dry weight of alga in such culture at that stage was 3–4 g L^−1^. At the end of each culture period, 400 ml of culture containing algal cells was placed in 1000 ml of new culture medium.

Algal growth was evaluated by measuring the specific growth rate. The specific growth rate μ was determined using the following formula: μ = ln (M_t2/_M_t1_)/(t_2_ - t_1_), where M_t2_ and M_t1_ denote the dry weight of the alga at time t_1_ and t_2_, respectively [19]. The dry weight was measured after the algal cells were filtered on a glass fiber filter (GF/A 110-mm diameter, Whatman), rinsed with deionized water and then dried at 105°C until the weight became constant.

### 2. Hydrocarbon Recovery Rate

Hydrocarbon recovery rate was calculated as the ratio of the amount of hydrocarbons extracted from the intact wet algal biomass to that extracted from the freeze-dried algal sample.

The quantity of hydrocarbons from the freeze-dried sample was determined using the following method. Two hundred milliliters of algal culture were freeze-dried. The freeze-dried sample was soaked in 50 ml of *n*-hexane at 25°C for 15 min followed by separation of the *n*-hexane extract containing hydrocarbons by decantation. Extraction was repeated usually four times until the *n*-hexane extracts were no longer colored with yellow pigments that co-existed with hydrocarbons in the extracellular matrix. The extracts were combined, concentrated using a rotary evaporator and subjected to silica gel column chromatography (Wakogel C-300, Wako Pure Chemical Industries) using *n*-hexane as a mobile phase to obtain a pure hydrocarbon fraction. All eluates from the column before the elution of carotenes that could be recognized as a yellow band were collected as a hydrocarbon fraction. The hydrocarbon fraction was collected in a pre-weighed flask and solvent was removed by a rotary evaporator. The residual oil in the flask was further dried in a vacuum desiccator for one hour and its weight was measured gravimetrically as the amount of extracellular hydrocarbon.

The quantity of hydrocarbons extracted from wet samples of the alga without pretreatment was determined using the following method. 200 ml of algal culture were transferred to a separation funnel with 200 ml of *n*-hexane. The funnel was vigorously shaken by hand for 30 s and then settled until two phases were separated. The upper yellow organic phase containing hydrocarbons was separated from the aqueous phase containing algal cells, and transferred into a flask. The aqueous phase was returned to the funnel again with 100 ml of *n*-hexane and similarly shaken for 30 s. The second organic phase was removed and combined with the first one, and concentrated by a rotary evaporator. The residual oil was handled in the same way as mentioned above for the hydrocarbons from the freeze-dried algal sample, and the amount of extracellular hydrocarbons extracted from the wet algal biomass without pretreatment was determined.

## Results and Discussion

### 1. Effect of Salinity in Culture Media on Hydrocarbon Recovery

It was found that hydrocarbon was extracted more readily from *B. braunii* cultured in the 1/4 seawater medium compared with those cultured in a freshwater medium. Then we examined what concentration of ASWM is effective to make living *B. braunii* susceptible to such easy hydrocarbon extraction.

**Figure 1:**
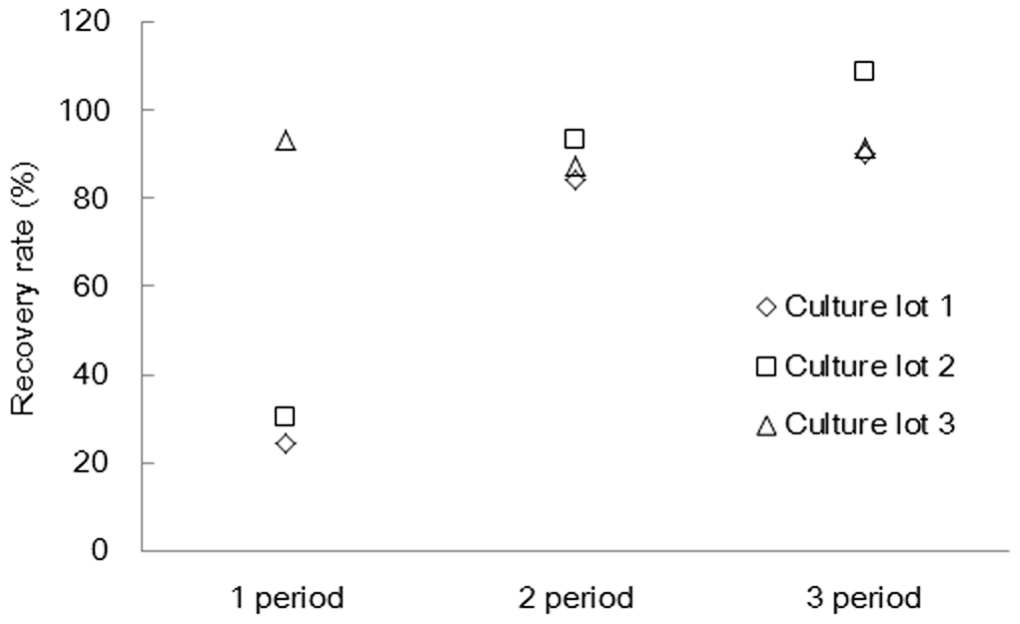
Changes in hydrocarbon recovery rate during culture in the 1/4 ASWM. The alga was cultured in 1/4 ASWM and the subculture was run three times. Different symbols represent different culture lots.

The algae were cultured in each medium prepared with artificial seawater, in which salinity was adjusted to 1/1, 1/2, and 1/4 ASWM, respectively. Algal death was, however, observed in the 1/1 and 1/2 ASWM, but an increase in algal biomass was observed in the 1/4 ASWM. Therefore we tried to know how long culture in 1/4 ASWM is necessary. Figure 1 shows the changes in hydrocarbon recovery rate from algae cultured in the 1/4 ASWM over three culture periods. The alga cultured in the modified Chu13 was placed into new 1/4 seawater medium. Moreover this alga was placed into new medium after 35 days culture and the subculture was run three times. This operation was replicated at three culture lots. Average of hydrocarbon recovery rates from the alga cultured in the modified Chu13 was only 2.6% and was almost constant irrespective of culture periods. Even if the alga was cultured in the modified Chu13 with subcultured repeatedly over the long term, hydrocarbon was not able to be recovered from wet sample by hexane extraction. In contrast, hydrocarbon recovery rates of at least 20% were obtained from algae cultured in the 1/4 ASWM even after one culture period. These rates were much higher than those from the alga in the modified Chu13. After two culture periods, the hydrocarbon recovery rate from the alga cultured in 1/4 ASWM was at least 80%. After three culture periods, it became over 90%. Thus there seemed to be a tendency that the longer the alga was cultured in 1/4 ASWM, the more readily the hydrocarbon recovery could be recovered. A recovery rate of over 90% was, however, obtained in one lot after the first culture period. The reason for the exceptionally rapid change in hydrocarbon recovery in this lot is not clear currently, but it is possibly due to the difference of growth stage of the seed culture when inoculated into new medium or the difference in growth rate among different lots. Kita et al. reported a high hydrocarbon recovery such as 86.7% from wet cells of the Showa strain cultured in the Chu13 medium by a simple solvent partition with n-hexane [11]. It was, however, achieved only after the algal samples had been pre-heated at 85°C before extraction. This study showed that culture of *B. braunii* in 1/4 ASWM for longer than 70 days allowed high hydrocarbon recovery from wet algal cells without any pre-heating. This is potentially advantageous if hydrocarbons can be effectively extracted from living *B. braunii* in the culture medium without any damage due to heat-treatment.

**Figure 2:**
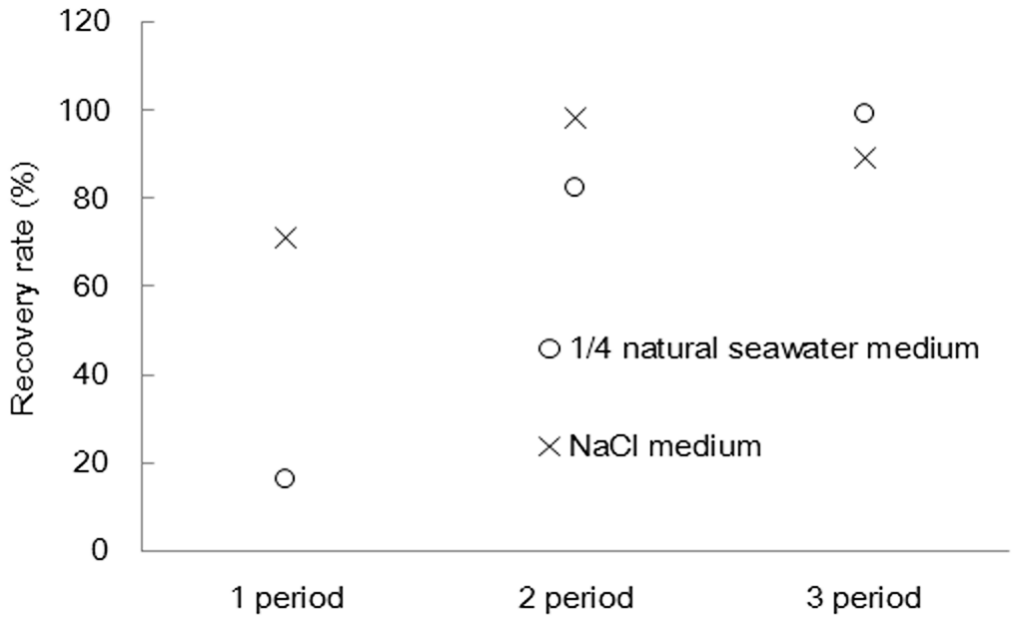
Changes in hydrocarbon recovery rate during culture in the 1/4 NSWM or the NaCl medium. Each alga was cultured in 1/4 NSWM or NaCl medium and the subculture was run three times.

As the artificial seawater enhanced recovery of hydrocarbons from wet algal cells of *B. braunii*, further experiments were carried out to confirm if the phenomenon was specifically-caused by artificial seawater medium or caused by osmotic shock with salts, especially sodium chloride, in the culture medium. Figure 2 shows the hydrocarbon recovery rate from the alga cultured in the 1/4 NSWM and the NaCl medium. After three culture periods, both types of medium achieved high hydrocarbon recovery rates, and the results showed a similar trend to that for the 1/4 ASWM. Therefore, there is a possibility that the increase in osmotic pressure by salt addition made it possible to recover over 90% of the hydrocarbon from the alga without pretreatment.

**Figure 3:**
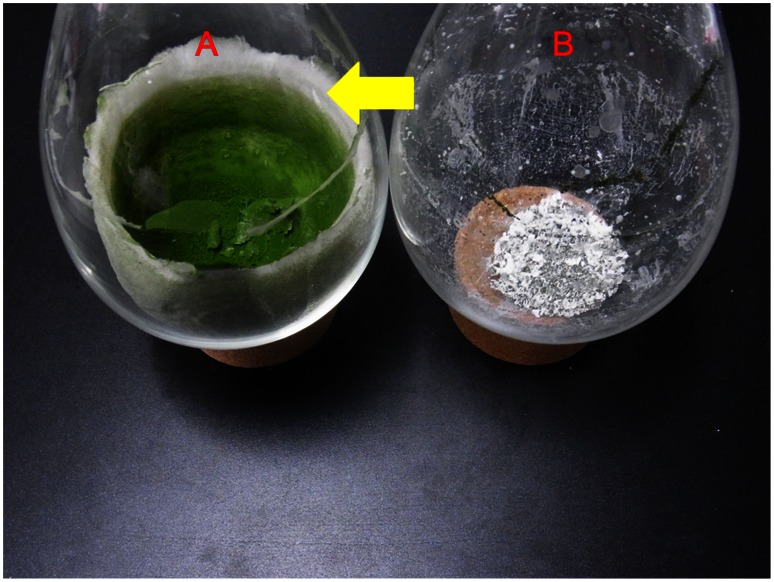
Difference in appearance of freeze-dried alga sample. Each sample was cultured in (A) the modified Chu13 medium and (B) 1/4 ASWM after three culture period. Dried algal samples cultured in the modified Chu 13 medium are usually covered with white furry matters (arrow) that can not be seen in one cultured in 1/4 ASWM.

Figure 3(A) shows the freeze-dried alga cultured in the modified Chu13. A furry matter that seems to be originated from secreted polysaccharides from the alga was present. On the other hand, Figure 3(B) shows the freeze-dried alga cultured in 1/4 ASWM after three culture periods in which there was no such furry matter. This decrease of furry matter is suspected to be due to decreasing of the secreted polysaccharides or shifts of their molecular sizes to smaller ones by osmotic pressure. It is hypothesized that the alga started de novo synthesis of more osmolytes to compensate for the osmotic shock and retarded production of polysaccharides or other macromolecules in the extracellular matrix instead. As polysaccharides or other biopolymers existing on the surfaces of colonies may play a role in sequestering the hydrocarbons in the colonies [12], it is suggested that decreasing of the surrounding polysaccharides or easier removal of the furry matters with much smaller molecular sizes from the colony weakened the extracellular matrix in which the hydrocarbons are stored, and made the hydrocarbon recovery process easier.

### 2. Effect of Artificial Seawater Medium on Growth

**Figure 4:**
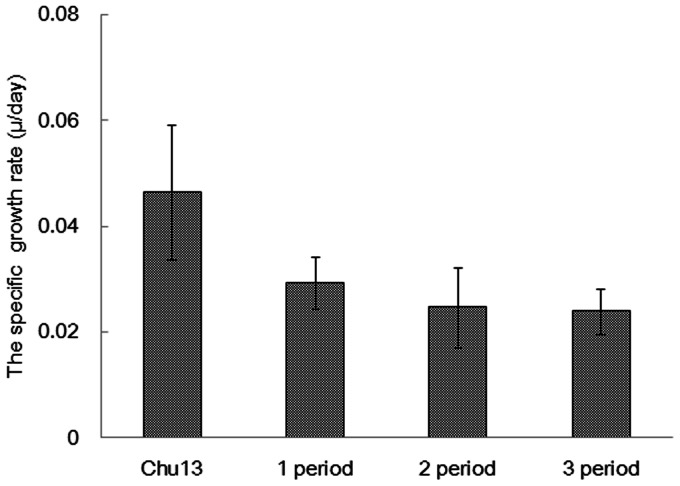
Specific growth rate (μ/day) of algae cultured in modified Chu13 medium and 1/4 ASWM. Data represents an average of three lots. Bars indicate mean ± SD.

**Figure 5:**
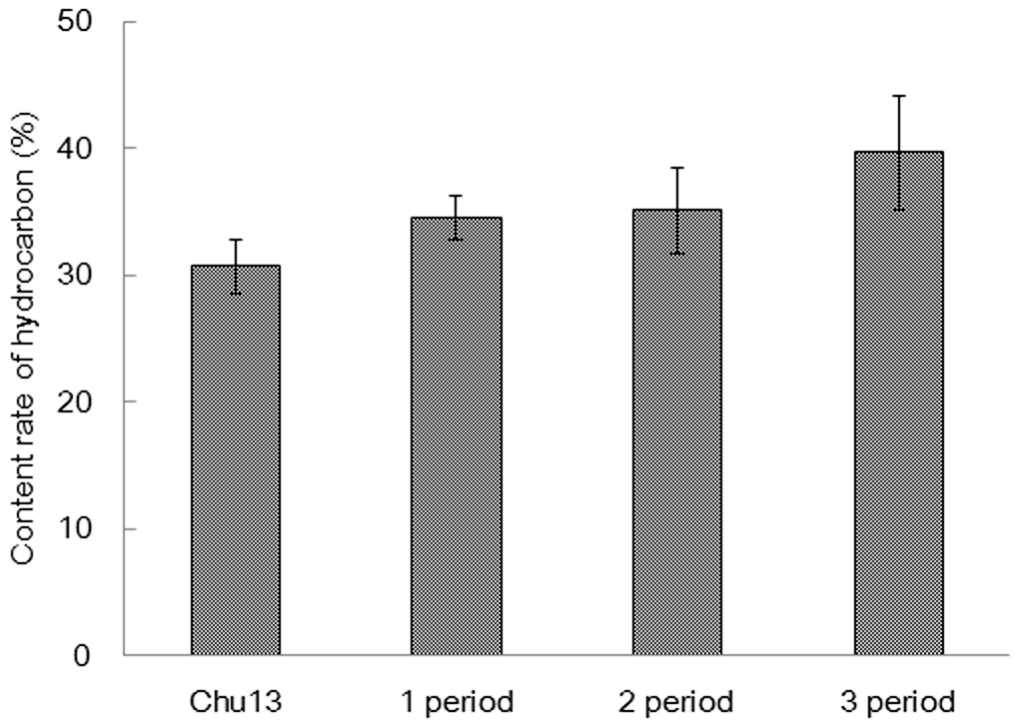
Hydrocarbon content (%) in dry biomass weight for algae. Algae was cultured in the modified Chu13 medium and 1/4 ASWM. Data represents an average of three lots. Bars indicate mean ± SD.

The growth rate of *B. braunii* is low due to the production of high energy hydrocarbon. The doubling generation time of *B. braunii* is longer than other microalgae [20]. Figure 4 shows the specific growth rate in the 1/4 ASWM. The growth slowed down by half due to osmotic stress (or high salinity). Other studies have also reported that the growth of *B. braunii* in a salt-added medium was slower than that in freshwater medium such as modified Chu13 [14], [21], [22]. By contrast, the hydrocarbon content in the dry biomass tended to increase, as shown in Fig. 5: modified Chu13 30.7%, first period in 1/4 ASWM 34.5%, second period 35.1%, and third period 39.6%, averaged over three lots. These results clearly confirmed that the microalga produced hydrocarbon, but the growth was inhibited.

This study revealed that culture of *B. braunii* in seawater enabled efficient hydrocarbon recovery by simply mixing intact wet alga with *n*-hexane. Moreover, the hydrocarbon content in the alga tended to increase by using a seawater medium. However, the algal growth rate was reduced to about half, which means a decrease in total hydrocarbon production from *B. braunii*. Thus, if it were possible to maintain the hydrocarbon recovery rate and achieve a growth rate equal to that of the alga cultured in freshwater by some method, for example, lowering the salinity in the medium or creating a salt-tolerant strain by breeding, it may be more advantageous to culture in seawater and omit pretreatment such as drying and heating, which consumes energy. This brings the possibility of cost reduction in hydrocarbon production from *B. braunii.* Additionally, omitting pretreatment avoids the necessity for cell rupture of *B. braunii* and suggests the potential for application of the milking process, which is a way to continuously extracting products from algae without destruction of the cells [23]. The ability to continuously utilize the biomass will also reduce the cost and energy required to propagate the algal.

### Conclusions

The Showa strain of *B. braunii* was cultured in a medium derived from the modified Chu13 medium by supplying artificial seawater for three culture periods, and a hydrocarbon recovery rate exceeding 90% was obtained. In the verification experiment, the results for the alga cultured in natural seawater medium or NaCl medium showed a similar trend to that for the alga cultured in artificial seawater medium. Therefore, it was suggested that the increase in osmotic pressure by salt addition made it possible to recover over 90% of the hydrocarbon from the alga even without pretreatment.
